# Demographic and Prescribing Patterns of Chinese Herbal Products for Individualized Therapy for Ischemic Heart Disease in Taiwan: Population-Based Study

**DOI:** 10.1371/journal.pone.0137058

**Published:** 2015-08-31

**Authors:** Yu-Chiang Hung, Ying-Jung Tseng, Wen-Long Hu, Hsuan-Ju Chen, Tsai-Chung Li, Pei-Yuan Tsai, Hsin-Ping Chen, Meng-Hsuan Huang, Fang-Yen Su

**Affiliations:** 1 Department of Chinese Medicine, Kaohsiung Chang Gung Memorial Hospital and School of Traditional Chinese Medicine, Chang Gung University College of Medicine, No.123, Dapi Rd., Niaosong Dist., Kaohsiung 833, Taiwan; 2 School of Chinese Medicine for Post Baccalaureate, I-Shou University, No.1, Sec. 1, Syuecheng Rd., Dashu District, Kaohsiung City 84001, Taiwan; 3 Fooyin University College of Nursing, No.151, Chinhsueh Rd., Ta-liao Dist., Kaohsiung City 831, Taiwan; 4 Kaohsiung Medical University College of Medicine, No.100, Shihcyuan 1st Rd., Sanmin Dist., Kaohsiung City 807, Taiwan; 5 Management Office for Health Data, China Medical University Hospital, No.2 Yude Road, Taichung 40447, Taiwan; 6 College of Medicine, China Medical University, No.91, Hsueh-Shih Road, Taichung 40402, Taiwan; 7 Graduate Institute of Biostatistics, College of Public Health, China Medical University, No.91, Hsueh-Shih Road, Taichung 40402, Taiwan; 8 Department of Healthcare Administration, College of Health Science, Asia University, No.500, Lioufeng Rd., Wufeng, Taichung 41354, Taiwan; The National Institute for Health Innovation, NEW ZEALAND

## Abstract

**Objective:**

Combinations of Chinese herbal products (CHPs) are widely used for ischemic heart disease (IHD) in Taiwan. We analyzed the usage and frequency of CHPs prescribed for patients with IHD.

**Methods:**

A nationwide population-based cross-sectional study was conducted, 53531 patients from a random sample of one million in the National Health Insurance Research Database (NHIRD) from 2000 to 2010 were enrolled. Descriptive statistics, the multiple logistic regression method and Poisson regression analysis were employed to estimate the adjusted odds ratios (aORs) and adjusted risk ratios (aRRs) for utilization of CHPs.

**Results:**

The mean age of traditional Chinese medicine (TCM) nonusers was significantly higher than that of TCM users. Zhi-Gan-Cao-Tang (24.85%) was the most commonly prescribed formula CHPs, followed by Xue-Fu-Zhu-Yu-Tang (16.53%) and Sheng-Mai-San (16.00%). The most commonly prescribed single CHPs were Dan Shen (29.30%), Yu Jin (7.44%), and Ge Gen (6.03%). After multivariate adjustment, patients with IHD younger than 29 years had 2.62 times higher odds to use TCM than those 60 years or older. Residents living in Central Taiwan, having hyperlipidemia or cardiac dysrhythmias also have higher odds to use TCM. On the contrary, those who were males, who had diabetes mellitus (DM), hypertension, stroke, myocardial infarction (MI) were less likely to use TCM.

**Conclusions:**

Zhi-Gan-Cao-Tang and Dan Shen are the most commonly prescribed CHPs for IHD in Taiwan. Our results should be taken into account by physicians when devising individualized therapy for IHD. Further large-scale, randomized clinical trials are warranted in order to determine the effectiveness and safety of these herbal medicines.

## Introduction

Ischemic heart disease (IHD) is the major contributor to the morbidity and mortality associated with coronary artery disease in the United States [1], Taiwan [2], and China [3]. According to a WHO report [4], an estimated 17.3 million people died from cardiovascular diseases (CVDs) in 2008, representing 30% of all global deaths. Of these deaths, an estimated 7.3 million were due to coronary heart disease. The number of people who die from CVDs will reach 23.3 million by 2030, 45% of which will be attributed to coronary heart disease [4]. In Taiwan, 11.5% of all deaths in 2013 were the result of heart diseases (excluding hypertension), and this percentage is growing. Heart disease was the second most common causes of death in Taiwan [2,5]. Therefore, research on IHD may provide valuable information for public health policy to government health institutions and other global health-research institutions.

Percutaneous coronary intervention, coronary artery bypass grafting, and some classes of drugs are commonly used in the treatment of IHD, such as beta blockers, calcium channel blockers, nitrates, ranolazine, and aspirin [6]. However, some medication are expensive and pose a heavy financial burden on low- and middle-income families [7]. Some complementary and alternative therapies may be available for major cardiac disorders [8]. Traditional Chinese medicine (TCM), especially combined herbal formulations, has been the most commonly used alternative therapy for cardiovascular disease in China for thousands of years [9]. Interest in complementary and alternative medicine is increasing, not only in patients seeking help, but also in researchers investigating the effectiveness of various therapies and interventions. Because patients with IHD have different clinical manifestations or syndromes, physicians treat them with different formulas of Chinese herbs.

Since 1995, Chinese herbal products (CHPs) have been listed under the National Health Insurance (NHI) program in Taiwan. The NHI database has provided unprecedented opportunities to access and analyze the prevalence and pattern of CHPs utilization in the general population [10,11]. Therefore, the aim of this study was to analyze the utilization of CHPs in patients with IHD in Taiwan in a population-based study, specifically. This study explored the demographic and prescribing patterns of CHPs in patients with IHD. The data will provide relevant research materials for clinical pharmacy and epidemiological studies. In addition, analysis of prescribing patterns will provide information regarding monitoring, evaluation, and modification of medical services and will establish some references for individualized therapy for IHD.

## Methods

### Data resources

This population-based study aimed to analyze the demographic and prescribing patterns of CHPs in patients with IHD derived from a random sample of one million beneficiaries in the National Health Insurance (NHI) program in Taiwan. Taiwan’s government launched the NHI program on March 1, 1995, and 22.60 million of the 22.96 million total population was enrolled in this program in 2007. To protect individual’s privacy, the data on patient identities were scrambled cryptographically by the National Health Insurance Research Database (NHIRD). Every individual in Taiwan has a unique personal identification number (PIN). All NHI datasets can be interlinked with each individual’s PIN. This study used the registry datasets for beneficiaries from 2000 to 2010 to examine outpatient care by visits, inpatient care by admissions, and ambulatory care orders. Prescription information was identified from the database for ambulatory care orders, including corresponding prescriptive orders and CHPs. Utilization of TCM outpatient services was defined as at least one TCM use, and all TCM care was provided in ambulatory clinics under NHI coverage. This study was conducted after approval by the Institutional Review Board of China Medical University in Central Taiwan (CMU-REC-101-012).

#### Study subjects

**Fig 1:**
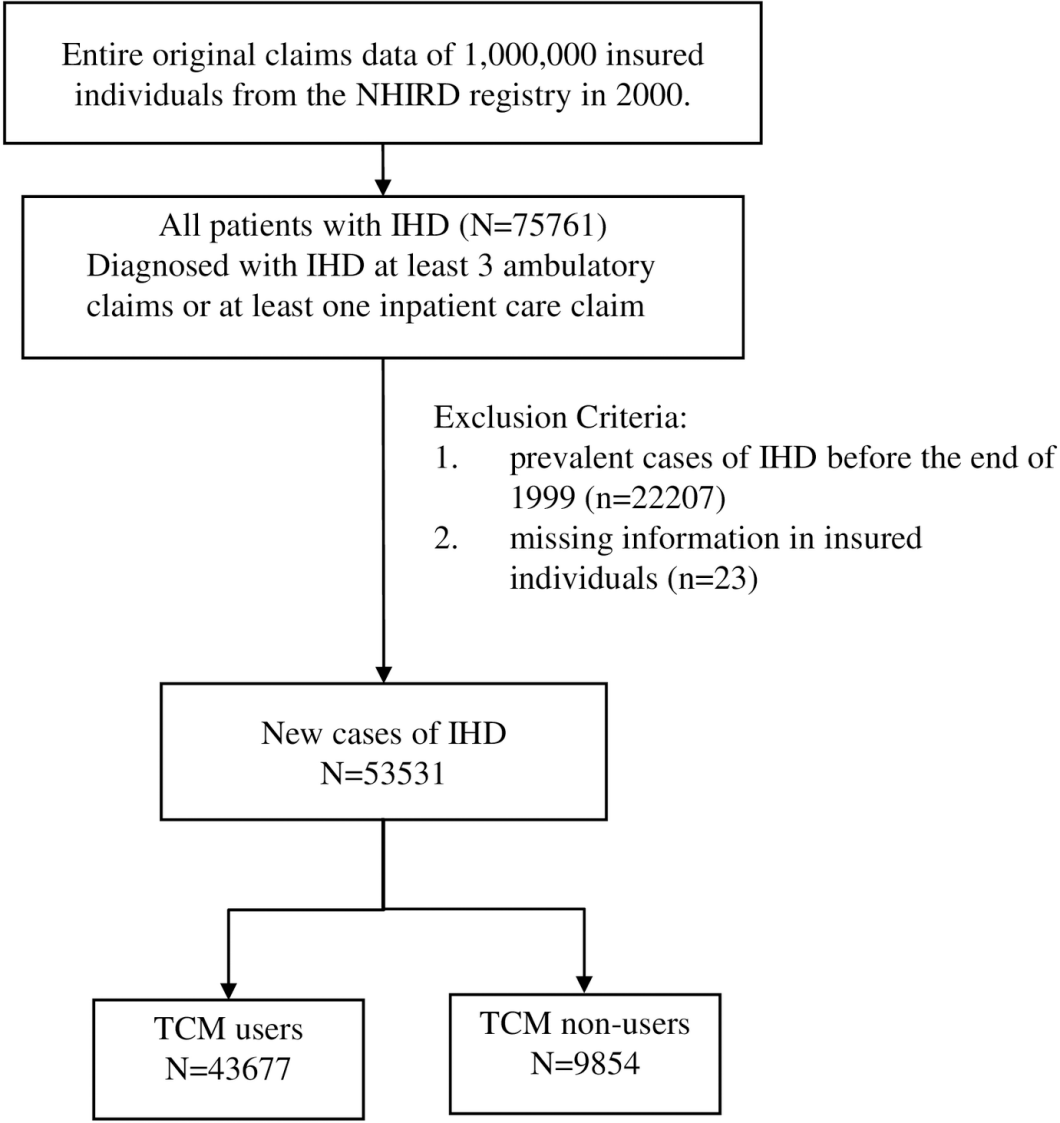
Flowchart of recruitment of subject recruitment from the 1-million random sample of the National Health Insurance Research Database (NHIRD) from 2000 to 2010 in Taiwan. Abbreviation: IHD, ischemic heart disease; TCM, traditional Chinese medicine.

We selected patients with IHD and a diagnosis code of 414, one of three major diagnosis codes according to the *International Classification of Diseases*, *Ninth Revision*, *Clinical Modification* (ICD-9-CM). The patients were selected from the random sample of one million individuals in the NHI dataset; Fig 1 depicts a flowchart of the recruitment process. We identified 75,761 patients diagnosed with IHD (ICD-9-CM code: 414) from the registries of outpatient care by visits and inpatient care by admission. We excluded patients with prevalent IHD (n = 22,207) that had been diagnosed prior to the end of 1999 and those with missing information on age or sex (n = 23). Thus, the final cohort included 53531 patients. These were divided into two groups: 9854 TCM nonusers and 43677 TCM users, and the values of their mean age were 66.75 (SD = 13.5) and 61.82 (13.14) years, respectively. Given the fixed sample size of 53531, the level of absolute precision *d*, *that* specifies the width of the 95% confidence interval (CI) would be 0.26% under the assumption that the TCM use in IHD patients was 75%. Because common values for *d* are usually around ±5% for estimated proportions in the range of 20%-80%, the width of the 95% CI in the present study is small, indicating the size of sample provides high precision for estimating the prevalence of TCM use in patients with IHD.

#### Study variables

To determine the key independent variables for the use of CHPs among IHD patients, we selected the demographic factors of sex, age, occupational status, geographic area, and risk factors for IHD. The baseline sociodemographic characteristics were determined from ID Registry of NHIRD by extracting data that was closest to the first diagnosed date of IHD and comorbidity history was determined for each patient using outpatient or inpatient claims within two years prior to the first diagnosed date. Age was categorized into five groups: ≤29, 30–39, 40–49, 50–59, and ≥60 years. We split occupational status into three levels: white collar, blue collar, and other. Geographic areas of Taiwan were classified into the following four regions: Northern Taiwan, Central Taiwan, Southern Taiwan, and Eastern Taiwan and offshore islands. We considered DM (ICD-9-CM: 250), hyperlipidemia (ICD-9-CM: 272), hypertension (ICD-9-CM: 401 to 405), cardiac dysrhythmias (ICD-9-CM: 427), stroke (ICD-9 CM: 430–438) and MI (ICD-9 CM: 410) as risk factors for IHD.

### Statistical analysis

Data analysis included the prevalence of TCM use stratified by the patient’s demographic and risk factors, frequency and proportion of the most frequently prescribed herbal formulas for treating IHD. A multiple logistic regression model was developed to estimate demographic and risk factors that correlated with TCM use. The models produced odds ratios (ORs) and corresponding 95% CIs. An adjusted odds ratio was used to predict patients who may have higher odds to use TCM therapy. The exposure period for counts of CHP or TCM use was defined as the period from the first diagnosed date to the date of withdrawal from the NHI program, death or the end of 2010. Risk ratios (RRs) and 95% confidence intervals (CI) were estimated for yearly counts of CHP by using Poisson regression analysis and sex, age, area, occupational status, DM, hyperlipidemia, hypertension, dysrhythmias, stroke, and MI were adjusted. All statistical analyses were performed using SAS 9.3 (SAS, Cary, NC, USA), with the significance level set to 0.05, two-tailed.

## Results

**Fig 2:**
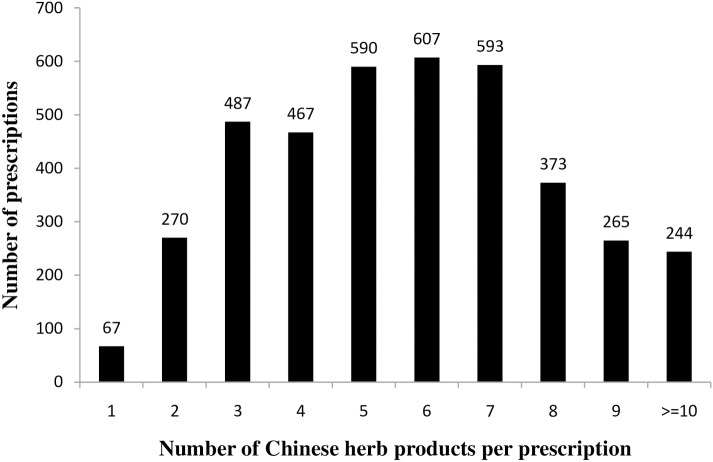
Distribution of the number Chinese herbal products in one prescription.

Of the 53551 patients with newly diagnosed IHD, 43677 (81.59%) used TCM outpatient services at least once. For patients with IHD who used TCM, there was an average of 5.67 Chinese herbs in a single prescription. The most commonly prescribed CHP combinations contained six (15.32%), seven (14.96%), and five (14.89%) CHPs in a single prescription (see Fig 2). Zhi-Gan-Cao-Tang (24.85%) was the most commonly prescribed formula CHP for patients with IHD, followed by Xue-Fu-Zhu-Yu-Tang (16.53%), Sheng-Mai-San (16.00%), and Tian-Wang-Bu-Xin-Dan (12.01). When only single CHPs were prescribed, the most common herbs were Dan Shen (29.30%), Yu Jin (7.44%), Ge Gen (6.03%), Huang Qi (5.60%), and Yan Hu Suo (5.37%). (See Table 1)

**Table 1:** The top 10 formulas and single CHPs prescribed by TCM physicians for treating patients with IHD from 2000 to 2010 in Taiwan (total prescription number: n = 3,963).

Formula CHPs	Number (%)	Single CHPs	Number (%)
Zhi-Gan-Cao-Tang	985 (24.85)	Dan Shen (*Salviae Miltiorrhizae*)	1,161 (29.30)
Xue-Fu-Zhu-Yu-Tang	655 (16.53)	Yu Jin (*Curcumaer*)	295 (7.44)
Sheng-Mai-San	634 (16.00)	Ge Gen (*Puerariae Lobatae)*	239 (6.03)
Tian-Wang-Bu-Xin-Dan	476 (12.01)	Huang Qi (*Astragali*)	222 (5.60)
Yang-Xin-Tang	347 (8.76)	Yan Hu Suo (*Corydalis*)	213 (5.37)
Jia-Wei-Xiao-Yao-San	327 (8.25)	Yuan Zhi (*Polygalae*)	190 (4.79)
Ma-Zi-Ren-Wan	216 (5.45)	Ye Jiao Teng (*Polygoni Multiflori*)	169 (4.26)
Qi-Ju-Di-Huang-Wan	202 (5.10)	Mai Men Dong (*Ophiopogonis*)	169 (4.26)
Bu-Yang-Huan-Wu-Tang	160 (4.04)	Du Zhong (*Eucommiae*)	165 (4.16)
Ban-Xia-Bai-Zhu-Tian-Ma- Tang	157 (3.96)	San Qi (*Notoginseng*)	161 (4.06)

Combination therapies were commonly prescribed for IHD patients. Zhi-Gan-Cao-Tang with Tian-Wang-Bu-Xin-Dan (4.67%) was the most commonly used two-formula-CHP combination, followed by Zhi-Gan-Cao-Tang with Xue-Fu-Zhu-Yu-Tang (4.29%), and Zhi-Gan-Cao-Tang with Yang-Xin-Tang (3.28%). In addition, the most commonly prescribed two-single-CHP for IHD was Dan Shen with Yu Jin (5.00%), followed by Dan Shen with Ge Gen (3.31%), and Dan Shen with Huang *Qi* (2.85%). (See Table 2)

**Table 2:** The top five most frequent combinations of CHPs pairs for IHD in Taiwan during 2000 to 2010 (total prescription number: n = 3,963).

Two-formula-CHPs	Number (%)	Two-single-CHPs	Number (%)
Zhi-Gan-Cao-Tang & Tian-Wang-Bu-Xin-Dan	185 (4.67)	Dan Shen & Yu Jin	198 (5.00)
Zhi-Gan-Cao-Tang & Xue-Fu-Zhu-Yu-Tang	170 (4.29)	Dan Shen & Ge Gen	131 (3.31)
Zhi-Gan-Cao-Tang & Yang-Xin-Tang	130 (3.28)	Dan Shen & Huang Qi	113 (2.85)
Xue-Fu-Zhu-Yu-Tang & Sheng-Mai-San	105 (2.65)	Dan Shen & Hong Hua	97 (2.45)
Zhi-Gan-Cao-Tang & Sheng-Mai-San	103 (2.60)	Dan Shen & Yuan Zhi	84 (2.12)

The TCM use differed depending on the age, sex, geographic area, occupation, or underlying risk factors of the patients. IHD Patients who aged 59 years and younger, who resided in Central Taiwan, who had hyperlipidemia, and who had cardiac dysrhythmias had higher odds to use TCM. However, IHD patients who were males, who resided in Eastern Taiwan and offshore islands, who had DM, who had hypertension, who had stroke, and who had MI were less likely to be TCM users (Table 3). The multivariate-adjusted odds ratio (aOR) and 95% CI resulting from the multiple logistic regression models are displayed in Table 3. P values for the overall test indicated that sex, age, occupational status, area, DM, hyperlipidemia, hypertension, cardiac dysrhythmias, stroke, and MI were significant factors associated with TCM use (Table 3). Compared with females, had lower odds to be TCM users (aOR = 0.52, 95% CI: 0.50 to 0.55). Compared to IHD patients over the age of 60 years, patients 59 years and younger had higher odds to use TCM (aOR >1). Especially, the odds using TCM for IHD patients under the age of 29 years was 2.62 times compared to IHD patients older than 60 years. While there was an overall significant effect of occupation status (p<0.001), there was no significant difference based on whether the patient was classified as a white- or blue-collar worker (aOR = 0.99, 95% CI: 0.93 to 1.04). In addition, patients registered in the Central Taiwan region had higher odds to be TCM users than those registered in the Northern Taiwan region (aOR = 1.58, 95% CI: 1.48 to 1.69). In contrast, patients registered in the Eastern Taiwan and offshore islands regions were less likely to be TCM users (aOR = 0.90, 95% CI: 0.82 to 0.98). In the multiple logistic regression models of comorbidity, IHD patients with DM, hypertension, stroke, or MI were less likely to be TCM users (aOR = 0.89, 95% CI: 0.85 to 0.94 for DM; aOR = 0.93, 95% CI: 0.88 to 0.98 for hypertension; aOR = 0.68, 95% CI: 0.63 to 0.73; and aOR = 0.80, 95% CI: 0.74 to 0.85), and those with hyperlipidemia or cardiac dysrhythmias had higher odds to be TCM users (aOR = 1.47, 95% CI: 1.40 to 1.55 for hyperlipidemia; aOR = 1.12, 95% CI: 1.06 to 1.19 for cardiac dysrhythmias).

**Table 3:** Demographic characteristics and results of multiple logistic regression models showing the adjusted odds ratios (OR) and 95% confidence intervals (CIs) of patients with ischemic heart disease from 2000 to 2010 in Taiwan.

Characteristics	TCM nonuser		TCM user		p-value	Crude OR (95% CI)	Adjusted OR (95% CI)	#p-value for overall effect
	N	%	N	%				
Number of cases	9854	18.41	43677	81.59				
Sex					<0.001			<0.001
Female	3375	13.25	22090	86.75		1.00	1.00	
Male	6479	23.08	21587	76.92		0.51 (0.49–0.53)***	0.52 (0.50–0.55)***	
Age (years)					<0.001			<0.001
≤29	49	9.55	464	90.45		2.64 (1.96–3.54)***	2.62 (1.94–3.54)***	
30–39	223	11.91	1650	88.09		2.06 (1.79–2.37)***	2.07 (1.79–2.40)***	
40–49	972	13.55	6202	86.45		1.78 (1.65–1.91)***	1.69 (1.56–1.82)***	
50–59	1780	14.13	10819	85.87		1.69 (1.60–1.79)***	1.56 (1.47–1.65)***	
≥60	6830	21.77	24542	78.23		1.00	1.00	
Mean (SD)	66.75	(13.49)	61.82	(12.14)	<0.001			
Occupational status					<0.001			<0.001
White collar	3362	16.76	16694	83.24		1.00	1.00	
Blue collar	3946	17.20	18991	82.80		0.97 (0.92–1.02)	0.99 (0.93–1.04)	
Other	2546	24.16	7992	75.84		0.63 (0.60–0.67)***	0.78 (0.73–0.82)***	
Area					<0.001			<0.001
Northern Taiwan	4427	19.62	18136	80.38		1.00	1.00	
Central Taiwan	1574	13.73	9890	86.27		1.53 (1.44–1.63)***	1.58 (1.48–1.69)***	
Southern Taiwan	3151	19.30	13173	80.70		1.02 (0.97–1.07)	1.05 (0.99–1.10)	
Eastern Taiwan and offshore islands	702	22.08	2478	77.92		0.86 (0.79–0.94)**	0.90 (0.82–0.98)*	
Risk factors								
DM					<0.001			<0.001
No	7289	18.02	33150	81.98		1.00	1.00	
Yes	2565	19.59	10527	80.41		0.90 (0.86–0.95)***	0.89 (0.85–0.94)***	
Hyperlipidemia					<0.001			<0.001
No	6431	20.60	24785	79.40		1.00	1.00	
Yes	3423	15.37	18892	84.66		1.43 (1.37–1.50)***	1.47 (1.40–1.55)***	
Hypertension					<0.001			0.009
No	2300	16.29	11822	83.71		1.00	1.00	
Yes	7554	19.17	31855	80.83		0.82 (0.78–0.86)***	0.93 (0.88–0.98)**	
Cardiac dysrhythmias					0.08			<0.001
No	8036	18.55	35280	81.45		1.00	1.00	
Yes	1818	17.80	8397	82.20		1.05 (0.99–1.11)	1.12 (1.06–1.19)***	
Stroke					<0.001			<0.001
No	8494	17.51	40014	82.49		1.00	1.00	
Yes	1360	27.08	3663	72.92		0.57 (0.53–0.61)***	0.68 (0.63–0.73)***	
MI					<0.001			
No	8545	17.82	39400	82.18		1.00	1.00	<0.001
Yes	1309	23.43	4277	76.57		0.71 (0.66–0.75)***	0.80 (0.74–0.85)***	

Abbreviation: TCM, traditional Chinese medicine; OR, odds ratio; CI, confidence interval; DM, diabetes mellitus; MI, myocardial infarction.

Adjusted OR#: mutually adjusted for sex, age, occupational status, area, diabetes mellitus, hyperlipidemia, hypertension, cardiac dysrhythmias, stroke, and myocardial infarction in the logistic regression model.

*p < 0.05

**p < 0.01

***p < 0.001.

The multivariate-adjusted risk ratio (aRR) and 95% CI were further applied to explore yearly total number of prescriptions of CHP for TCM using the multiple Poisson regression models (Table 4). The multivariate-adjusted RR indicated that calendar year of diagnosed IHD, sex, age, occupational status, area, DM, hyperlipidemia, hypertension, cardiac dysrhythmias, stroke, and MI were significant factors associated with counts of CHP. The yearly incidence rate fluctuated instead of a linear trend. Patients with IHD in age groups of ≤29, 30–39, 40–49, and 50–59 years, who resided in areas of Central Taiwan, Southern Taiwan, and Eastern Taiwan and offshore islands, who had hyperlipidemia, and who had cardiac dysrhythmias were more likely to have higher counts of CHP than their counterparts. On the contrary, patients with IHD who were male, who were blue-collar workers, who had DM, who had hypertension, who had stroke, and who had MI were less likely to have higher counts of CHP than their counterparts.

**Table 4:** Demographic characteristics and results of multiple Poisson regression models of yearly total number of prescriptions of CHP for TCM showing the adjusted rate ratios (RR) and 95% confidence intervals (CIs) of patients with ischemic heart disease from 2000 to 2010 in Taiwan.

Characteristics	N	Total number of prescriptions of CHP for TCM	Rate#	Crude RR (95% CI)	p value for overall effect	Adjusted† RR (95% CI)	p value for overall effect
Calendar year of diagnosed IHD					<0.001		<0.001
2000	9316	127031	153.36	1.00		1.00	
2001	6981	87188	154.16	1.01 (0.99–1.01)		0.99 (0.98–0.99)*	
2002	5875	68624	159.31	1.04 (1.03–1.05)***		1.03 (1.02–1.04)***	
2003	5253	54307	156.31	1.02 (1.01–1.03)***		0.99 (0.99–1.01)	
2004	4946	44825	155.77	1.02 (1.00–1.03)**		1.00 (0.99–1.01)	
2005	4380	34888	160.70	1.05 (1.04–1.06)***		1.05 (1.04–1.06)***	
2006	3785	24757	157.92	1.03 (1.02–1.04)***		1.03 (1.01–1.04)***	
2007	3813	18681	148.23	0.97 (0.95–0.98)***		0.96 (0.94–0.97)***	
2008	3467	13054	157.81	1.03 (1.01–1.05)**		1.03 (1.01–1.05)***	
2009	3239	7473	158.89	1.04 (1.01–1.06)**		1.03 (1.01–1.06)**	
2010	2476	2014	147.57	0.96 (0.92–1.01)		0.98 (0.94–1.03)	
Sex					<0.001		<0.001
Female	25465	264120	170.56	1.00		1.00	
Male	28066	218722	140.70	0.82 (0.82–0.83)***		0.83 (0.82–0.83)***	
Age, years					<0.001		<0.001
≤29	513	6022	166.67	1.19 (1.16–1.22)***		1.03 (0.99–1.05)	
30–39	1873	23879	201.53	1.43 (1.42–1.45)***		1.33 (1.31–1.35)***	
40–49	7174	81906	182.59	1.30 (1.29–1.31)***		1.24 (1.23–1.25)***	
50–59	12599	124600	167	1.19 (1.18–1.20)***		1.15 (1.14–1.16)***	
≥60	31372	246435	140.51	1.00		1.00	
Occupational status					<0.001		<0.001
White collar	20056	184338	158.66	1.00		1.00	
Blue collar	22937	208102	156.67	0.99 (0.98–0.99)***		0.92 (0.92–0.93)***	
Others	10538	90402	147.47	0.93 (0.92–0.94)***		0.99 (0.99–1.01)	
Area					<0.001		<0.001
Northern Taiwan	22563	164715	126.04	1.00		1.00	
Central Taiwan	11464	135621	202.30	1.61 (1.59–1.62)***		1.64 (1.63–1.65)***	
Southern Taiwan	16324	157618	167.27	1.33 (1.32–1.34)***		1.37 (1.36–1.38)***	
Eastern Taiwan and offshore islands	3180	24888	135.53	1.08 (1.06–1.09)***		1.13 (1.11–1.15)***	
Risk factors							
DM					<0.001		<0.001
No	40439	383715	159.33	1.00		1.00	
Yes	13092	99127	142.65	0.90 (0.89–0.90)***		0.91 (0.90–0.92)***	
Hyperlipidemia					<0.001		<0.001
No	31216	275688	150.1	1.00		1.00	
Yes	22315	207154	163.58	1.09 (1.08–1.10)***		1.17 (1.16–1.18)***	
Hypertension					<0.001		0.01
No	14122	158977	186.7	1.00		1.00	
Yes	39409	323865	143.83	0.77 (0.77–0.78)***		0.81 (0.80–0.81)***	
Cardiac dysrhythmias					<0.001		<0.001
No	43316	384465	151.13	1.00		1.00	
Yes	10215	98377	175.91	1.16 (1.16–1.17)***		1.17 (1.16–1.18)***	
Stroke					<0.001		<0.001
No	48508	455559	157.81	1.00		1.00	
Yes	5023	27283	126.13	0.80 (0.80–0.81)***		0.87 (0.86–0.88)***	
MI					<0.001		<0.001
No	47945	445858	157.45	1.00		1.00	
Yes	5586	36984	136.3	0.87 (0.86–0.87)***		0.89 (0.88–0.90)***	

Abbreviation: RR, rate ratio; CI, confidence interval; IHD, ischemic heart disease; DM, diabetes mellitus; MI, myocardial infarction.

# per 100 person-years

†Adjusted for year of diagnosed ischemic heart disease, sex, age, occupational status, area, DM, hyperlipidemia, hypertension, cardiac dysrhythmias, stroke, and myocardial infarction.

*p < 0.05

**p < 0.01

*** p<0.001

## Discussion

This is an important, large-scale survey on the use of CHPs for the treatment of IHD that analyzed the dataset on use of TCM in outpatient-clinic visits covered by the NHI in Taiwan. We explored factors that may influence patients with IHD to seek TCM treatment, such as sex, age, occupation status, area of residence, and IHD risk factors. According to our literature review, this study is the first to use a random, thus population-based cross-sectional study to document the demographic and CHPs prescribing patterns for patients with IHD on a nationwide scale. Our results (see Table 3) showed that both sex and geographic area were relevant factors. Specifically, females had higher odds of being TCM users, which are consistent with those conducted by Liao et al in patients with lung cancer [12] or with liver cancer [13]. The possible explanation may be that females were more likely to seek for TCM, or that the sex effect was due to confounding effect by those factors not included in the regression models. In addition, compared to IHD patients in Northern Taiwan, the odds of TCM use was higher in Central Taiwan but lower in Eastern Taiwan and the offshore islands. This may be related to the distribution of TCM physicians. The average number of TCM physicians per ten thousand people was 3.59 in Central Taiwan, but only 2.27, 2.16, and 1.66 in Northern Taiwan, Southern Taiwan, and Eastern Taiwan, respectively. Thus, the distribution of TCM physicians may influence patients’ healthcare-seeking behavior. Our study shows that patients with IHD in Taiwan have a high level of TCM-seeking behavior, which may provide a reference for public health policymakers and clinicians in the area of allocation of medical resources.

In addition to statistical reports on the frequency of prescriptions, this study demonstrates that combinations of CHPs, such as Zhi-Gan-Cao-Tan and Dan Shen, play a significant role in prescriptions for IHD. These results should be taken into account by physicians when devising individualized therapy for IHD. CHPs are usually prescribed together as core formulae or couplet medicinals in order to enhance effectiveness or minimize toxicity; therefore these should be analyzed in combined patterns. The current study is capable of finding co-prescribed CHPs with high frequency in a large-scale nationwide prescription database. Our results are consistent with previous studies that have revealed the possible mechanisms of these formulas or herbal drugs (see Table 5). For example, patients with IHD suffering from palpitation with tachycardia or bradycardia could be treated with Zhi-Gan-Cao-Tang [14]. One previous study showed that Xue-Fu-Zhu-Yu-Tang has the potency to lower serum total-triglyceride concentration, strongly decrease the TXA2/PGI2 ratio, and attenuate production of pro-inflammatory cytokines in high-cholesterol-fed rats [15]. Another study indicated that Xue-Fu-Zhu-Yu-Tang inhibits ischemic myocardial apoptosis, most likely through a sensitization agent that inhibits SIRT1 (silent mating type information regulation 2 homolog-1) apoptosis pathways [16]. Sheng-Mai-San could reduce myocardial infarct size through activation of protein kinase C, opening of the mitochondrial KATP channel, and reducing oxidative damage [17–19]. Dan Shen has antioxidant effects, inhibits smooth-muscle-cell proliferation, and protects against vascular atherosclerotic lesions by circulating ROS suppression via the PKC/p44/42 MAPK-dependent pathway [20–25]. In addition, Danshen combined with Gegen has vasodilation, protects the myocardium against ischemia/reperfusion injury via the redox-sensitive PKCvarepsilon/mK (ATP) pathway, and inhibits mitochondrial permeability transition via the redox-sensitive ERK/Nrf2 and PKC epsilon/mKATP pathway [26–33]. Yu Jin may also have effects on CVD [34].

**Table 5:** Possible mechanisms of frequently used CHPs for IHD from 2000 to 2010 in Taiwan

	Known active herb constituents and formula ingredients	Possible pharmacological effects on IHD
Formula CHPs		
Zhi-Gan-Cao-Tang	*Radix Glycyrrhizae Preparata*, *Radix Ginseng*, *Ramulus Cinnamomi*, *Radix Rehmanniae*, *Tuber Ophiopogonis*, *Colla Corii Asini*, *Semen Cannabis*, *Rhizome Zingiberis Recens*, *Fructus Zizyphi Jujube*, *White Wine*.	Warms *Heart Yang*, improves blood circulation, and removes blood stasis [14].
Xue-Fu-Zhu-Yu-Tang	*Semen Persicae*, *Flos Carthami*, *Radix Angelicae Sinensis*, *Rhizome Chuanxiong*, *Radix Paeoniae Rubra*, *Radix Cyathulae*, *Radix Bupleuri*, *Radix Platycodi*, *Fructus Aurantii*, *Radix Rehmanniae*, *Radix Glycyrrhizae*.	Lowers the serum total-triglyceride concentration, decreases the TXA2/PGI2 ratio, and attenuates production of pro-inflammatory cytokines [15]. Inhibits the ischemic myocardial apoptosis [16].
Sheng-Mai-San	*Radix Ginseng*, *Tuber Ophiopogonis*, *Fructus Schisandrae*.	Activates protein kinase C and opens the mitochondrial KATP channel [17]. Attenuates myeloperoxidase activities and malondialdehyde levels, decreases superoxide dismutase activities, protects myocardial tissue, and reduces oxidative damage [18]. Protects against oxidative stress and tunes patients’ immune response [19].
Single CHPs		
Dan Shen (*Salviae Miltiorrhizae*)	Salvianolic acid, Rosmarinic acid, Magnesium tanshinoate B, Tanshinone.	Antioxidant [20–25]. Inhibits vascular contractions [21]. Inhibits smooth-muscle-cell proliferation and protects against vascular atherosclerotic lesions through circulating ROS suppression via the PKC/p44/42 MAPK-dependent pathway [23,24].
Yu Jin (*Curcumae*)	Foraerugidiol, Zedoarondiol.	Protective and therapeutic effects on cardiovascular and cerebrovascular diseases [34].
Ge Gen (*Puerariae Lobatae)*	Flavone extracts from Puerariae Radix	Lowers blood pressure and decreases cerebral vascular resistance [26,27]. Protects against myocardial ischemia/reperfusion injury [28,29]. Vasodilation or vasorelaxation [30–32]. Inhibits mitochondrial permeability transition via the redox-sensitive ERK/Nrf2 and PKC epsilon/mKATP pathway [33].

Optimal TCM treatments involve carefully chosen CHPs that are intended to work synergistically to harmonize the patient’s underlying condition. Patients with IHD had different predispositions to disease, disease development, natural course of IHD, and response to therapeutic intervention that are caused by an interaction between age, sex, genetic background, environmental factors, lifestyle, culture and beliefs, and social status. The prescription of CHPs may vary according to the expression of these factors.

Because the NHI system has comprehensive coverage for medical treatments including TCM prescriptions, almost all people in Taiwan have this insurance under the government's policy. While the NHIRD is a very complete database including 22.60 million of 22.96 million people, the representativeness of this database is reliable and strong to provide some information of the patients' medical seeking behavior and physicians' prescription patterns in Taiwan. In addition, advantage of our study merits attention. The completeness of NHIRD and nationwide population-based study design increase the representativeness of our study sample, which can prevent selection bias.

There are some limitations of our study. First, this study did not determine the effectiveness and mechanism of therapeutic effects. Despite that the NHIRD contains large amounts of prescription data, chart-level records (i.e., physician notes, laboratory reports, and imaging studies) were not available. Thus, it was not possible to evaluate the effectiveness of the treatments. Second, this study focused on CHPs that are certified by good manufacturing practice standards in Taiwan. Certified CHPs are available to the public with standardized constituents and dosages, which makes this study reproducible and comparable to other studies. However, there are some Chinese herbal remedies that can be purchased directly from TCM herbal pharmacies, and some health foods containing herbs do not fall within the categories investigated in this study. Thus, the frequency of CHP utilization might have been underestimated in this study. However, because the NHI system has comprehensive coverage for TCM prescriptions, which generally cost less than the herbs sold in Taiwan markets, the likelihood that patients purchased a large amount of other herbs outside the NHI database is low. Third, TCM users with IHD may have used western medicine as well as CHPs. For example, we know that Danshen interacts with warfarin by potentiating its anticoagulant action. We suggest that a more critical attitude toward the use of anticoagulants and CHPs in combination is needed among both TCM physicians and anticoagulant users. Further study is needed to investigate the population of patients using such combinations and the effectiveness of these combinations because potential herb-drug interactions may lead to unpredictable consequences. Fourth, the database does not contain information on education, marital status, smoking, alcohol consumption, and exercise, which may also be associated with TCM use.

## Conclusions

CHPs are commonly used for the treatment of IHD in Taiwan. Our study revealed that among TCM users with IHD, females, white-collar workers, those registered as living in Central Taiwan, and those with hyperlipidemia and dysrhythmias are the dominant population. Various CHPs that have particular effects are used synergistically to optimize the treatment of IHD. Our results found the core formula and commonly prescribed CHP combinations. Zhi-Gan-Cao-Tang and Dan Shen are the most frequently prescribed CHPs by TCM physicians in Taiwan for patients with IHD. These results provide information for individualized therapy for IHD. In addition, further well-conducted, double-blind, randomized, placebo-controlled studies are needed to evaluate the effectiveness and safety of these CHP combinations for IHD.
